# Supplementary material for: Qualitative study exploring which research outcomes best reflect women’s experiences of heavy menstrual bleeding: stakeholder involvement in development of a core outcome set
Source: BMJ Open. 2023 Jul 14;13(7):e063637. doi: 10.1136/bmjopen-2022-063637 (PMC10357648; doi:10.1136/bmjopen-2022-063637)
Supplement: Supplementary data [file bmjopen-2022-063637supp001.pdf]

Appendix 1. Workshop / interview question template

Facilitator notes – _Please write down the comments to the below questions in the notepads provided_

Opening

- Can you tell me one thing that particularly bothers you about this condition?
- Can you tell me one thing that you think could particularly bother *other* women with this condition?

Life at home and in general

- In a typical week during the course of treatment, is there anything in your life that has been affected by the condition that you would like to see improved?
- How do you think this represents women in general with HMB?
- How do you think this would differ if you were in a different group of women i.e., young women in their twenties or partners?

Activity of daily living (cleaning, cooking, shopping, walking)

- Does this condition stop you from doing normal daily activities?
- Does it affect your mood, sleep, appetite, mobility, bladder and bowel symptoms?
- Can you do chores like shopping, climbing stairs, cooking and doing the laundry?
- Are you in pain?
- What does the pain feel like?
- How does it affect your daily life?
- What makes it better i.e., pain medication, hot water bottle?

Life at work

- How does this condition affect your work life?
- If you were still working during the course of treatment, how would this affect your performance? – For example, a delayed return to work? Any loss of income?
- Do you feel you need additional help/aid around the house - either professional or personal help?

Relationship and family life

- How does this condition affect your relationship with your loved ones (partners, children, friends and relatives)?
- Is there any issue on intimacy with your partner?
- What aspects of the condition do you think he/she want to know more about?

Emotional, anxiety, stress levels

- Do you think it is useful in general to explore a way to measure the emotional and psychological wellbeing of yourself and loved ones during the course of treatment?

Summary, closure and most important point

- If I were to share your answers to the entire group at the workshop, what would be your most important point/outcome to highlight?
