# Supplementary material for: Qualitative study exploring which research outcomes best reflect women’s experiences of heavy menstrual bleeding: stakeholder involvement in development of a core outcome set
Source: BMJ Open. 2023 Jul 14;13(7):e063637. doi: 10.1136/bmjopen-2022-063637 (PMC10357648; doi:10.1136/bmjopen-2022-063637)
Supplement: Supplementary data [file bmjopen-2022-063637supp002.pdf]

Appendix 2. Table showing the organisation of transcript nodes and how they reflect research outcomes

Node from coding	Outcomes identified by literature review that correspond to the node
Adverse effects in general	Adverse events
Adverse effects on fertility	Adverse events<br>Degree of satisfaction with treatment
Anaemia	Haemoglobin value<br>Serum ferritin<br>Iron parameters
Anxiety	Anxiety<br>Psychological wellbeing
Appetite	Severity of nausea and vomiting<br>Weight change<br>Effect of menstruation on activities of every-day living
Available resources	Degree of satisfaction with treatment
Changing sanitary protection	Number of sanitary items used
Childcare	Effect of menstruation on activities of every-day living
Communication	Psychological wellbeing<br>Degree of satisfaction with treatment
Concentration	Effect of menstruation on professional activities or education
Dyspareunia	Number of women with dyspareunia
Effect on bladder	Bladder symptoms
Effect on Bowel	Bowel symptoms
Emotional support	Psychological wellbeing<br>Degree of satisfaction with treatment
Emotions	Depression<br>Anxiety<br>Psychological wellbeing<br>Mood swings
Employment and education	Effect of menstruation on professional activities or education
Fatigue	Haemoglobin value<br>Serum ferritin<br>Iron parameters<br>Effect of menstruation on activities of every-day living
Fear	Depression<br>Anxiety<br>Psychological wellbeing<br>Adverse events
Feeling different	Anxiety<br>Psychological wellbeing
Feeling weak	Haemoglobin value<br>Serum ferritin<br>Iron parameters<br>Effect of menstruation on activities of ever- day living
Femininity	Femininity

Freedom	Effect of menstruation on activities of every-day living
Frustration	Effect of menstruation on activities of every-day living<br>Psychological wellbeing<br>Degree of satisfaction with treatment
General Health	Health related quality of life
GP Inconsistency	Degree of satisfaction with treatment
Headaches	Severity of headache<br>Number of days with headache<br>Adverse events
Heavy bleeding	Number of women whose symptoms have not changed or have become worse<br>Severity of menstrual blood reported in groups of increasing amounts<br>Severity of menstrual blood loss reported with a numerical score to represent severity<br>Patient's subjective assessment of heaviness of menstrual blood loss<br>Number of days of heavy bleeding<br>Number of women passing blood clots during menstruation<br>Number of women with flooding
Helplessness	Depression<br>Anxiety<br>Psychological wellbeing
Home life	Health related quality of life<br>Degree of effect of menstrual disturbance on health-related quality of life<br>Effect of menstruation on activities of every-day living
Hopelessness	Depression<br>Anxiety<br>Psychological wellbeing<br>Degree of satisfaction with treatment
Irregularity;	Number of women with infrequent periods<br>Degree of regularity of menses<br>Number of women with intermenstrual bleeding
Isolation	Depression<br>Psychological wellbeing
Knowledge	Degree of satisfaction with treatment
Lack of understanding and support	FROM PROFESSIONALS- degree of satisfaction with treatment.<br>FROM PARTNER- NEW OUTCOME NODE
Leaking	Number of women with flooding
Life affected on non-menstruating days	Health related quality of life<br>Effect of menstrual disturbance on sexual activity<br>Number of days a woman is unable to leave the house due to menstruation<br>Effect of menstruation on activities of every-day living
Lifestyle	Effect of menstruation on activities of every-day living
Loneliness	Health related quality of life<br>Depression<br>Psychological wellbeing
Mental Challenge	Depression

	Anxiety<br>Psychological wellbeing<br>Effect of menstruation on professional activities or education
Mental Health	Depression<br>Anxiety<br>Psychological wellbeing
Mood swings	Mood swings
Nausea and vomiting	Severity of nausea and vomiting
Pain	Pain during procedure<br>Severity of postoperative<br>Severity of pain at discharge from hospital<br>Duration of post- op pain<br>Severity of back pain<br>Severity of period pain<br>Number of women with period pain<br>Number of days with period pain<br>Severity of pain<br>Number of days on which pain experienced
Pain management	Use of painkillers<br>Number of women needing analgesia during procedure<br>Number of women needing post-op analgesia
Payoff between treatment and side effects	Degree of satisfaction with treatment<br>Adverse events
Practicalities or logistics	Number of sanitary items used<br>Number of days a woman is unable to leave the house due to menstruation<br>Effect of menstruation on activities of every-day living
Premenstrual Symptoms	Number of women with premenstrual symptoms<br>Severity of premenstrual symptoms
Prolonged bleeding	Duration of menses
Psychological effects	Psychological wellbeing
Restriction	Effect of menstruation on activities of every-day living<br>Number of days a woman is unable to leave the house due to menstruation
Scared	Anxiety<br>Psychological wellbeing
Sense of control	Psychological wellbeing<br>Degree of satisfaction with treatment
Sex	Effect of menstrual disturbance on sexual activity
Sleep disturbance	Severity of sleep disturbance
Suffering	Number of women whose symptoms have not changed or have become worse<br>Severity of menstrual blood loss<br>Severity of period pain<br>Severity of pain<br>Number of days on which pain experienced<br>Psychological wellbeing<br>Adverse events<br>Pain during procedure<br>Severity of postoperative pain

	Duration of post- op pain<br>Number of women needing analgesia during procedure<br>Number of women needing post-op analgesia
Treatment effectiveness	Patient's assessment of effectiveness of treatment<br>Number of women going on to have additional treatment for heavy periods
Treatment safety	Adverse events<br>Treatments specific safety measures
Treatment stopped	Discontinuation of treatment
**Nodes which do not correspond to outcomes identified from previous reported research**	
Cost to patient	No corresponding outcome
Partner inclusion	No corresponding outcome
Relationships	No corresponding outcome
Self-consciousness	No corresponding outcome
Self-Image	No corresponding outcome
Shame and embarrassment	No corresponding outcome
Stigma	No corresponding outcome
Understanding and empathy	No corresponding outcome
Lack of understanding / support	No corresponding outcome
**Excluded Nodes**	**Notes**
Age	EXCLUDED as not an outcome of treatment
Coping strategies	EXCLUDED as not an outcome of treatment
Delayed referral	EXCLUDED as not an outcome of treatment
Desire for fertility	EXCLUDED as not an outcome of treatment
Discrimination	EXCLUDED as not an outcome of treatment
Exercise	EXCLUDED as not an outcome of treatment
Fibroids	EXCLUDED as not an outcome of treatment
Government unsupportive	EXCLUDED as not an outcome of treatment
Hormonal treatments	EXCLUDED as not an outcome of treatment
Important outcomes	EXCLUDED as not an outcome of treatment
Nutrition	EXCLUDED as not an outcome of treatment
Outcome priority	EXCLUDED as not an outcome of treatment
Procedure complexity	EXCLUDED as not an outcome of treatment
Race	EXCLUDED as not an outcome of treatment
Religion	EXCLUDED as not an outcome of treatment
Responsibility	EXCLUDED as not an outcome of treatment
Seeking medical help	EXCLUDED as not an outcome of treatment
Self-reflection	EXCLUDED as not an outcome of treatment
Support Group	EXCLUDED as not an outcome of treatment
Treatment preference	EXCLUDED as not an outcome of treatment
